# Cyberaddiction and anxiety-depressive disorders among adolescents in Sousse, Tunisia: Prevalence, Patterns, and Implications

**DOI:** 10.1192/j.eurpsy.2025.929

**Published:** 2025-08-26

**Authors:** R. Ayoub, Y. Akkari, M. Ajmi, A. Lajnef, M. Oueslati, A. Guedria

**Affiliations:** 1 Child and Adolescent Psychiatry Department, Fattouma Bourguiba university Hospital; 2 University of Monastir, Monastir, Tunisia

## Abstract

**Introduction:**

As global internet usage continues to expand, concerns regarding its effects on adolescent mental health have gained prominence. Cyberaddiction and anxiety-depressive disorders are increasingly recognized as interconnected among adolescents. Excessive use of digital platforms, social media, and online gaming can contribute to heightened stress, social isolation, and emotional instability. Understanding these dynamics is essential for developing effective mental health strategies and encouraging healthier online behaviors among adolescents.

**Objectives:**

This study aimed to investigate the relationship between cyberaddiction and anxiety-depressive disorders among adolescents in Tunisia.

**Methods:**

This is a cross-sectional, descriptive, and analytical study, that was conducted on adolescents aged 12 to 18, enrolled in three educational institutions in Sousse, Tunisia. Data were collected using the Young Internet Addiction Scale and the Hospital Anxiety and Depression Scale (HADS). A pre-established data collection form was used to gather additional demographic and behavioral information.

**Results:**

Our study included a population of 416 adolescents, with a mean age of 15.05 ± 1.724 years. The majority of the participants were female (56.7%). Among the participants, 41.6% were high school students, and 58.4% were middle school students. Our results showed that 83.2% of participants exhibited problematic internet use, with potentially adverse effects on their daily lives. Additionally, 11.3% managed to maintain control over their internet usage despite occasionally exceeding planned time, while 5.5% experienced severe internet addiction, significantly impacting their personal and social lives Regarding internet usage patterns, the majority (64.9%) reported that social media was their primary online activity, while 35.1% preferred online gaming. The HADS revealed that 43% of the participants experienced varying degrees of anxiety disorders, and more than half (50.7%) showed symptoms of depressive disorders. Notably, 88.5% of the participants displayed symptoms of both anxiety and depression. A statistically significant correlation was observed between anxiety-depressive disorders and cyberaddiction (p = 0.008).

**Conclusions:**

Our study highlights a concerning prevalence of both cyberaddiction and anxiety-depressive disorders among adolescents. These findings emphasize the urgent need for preventive measures and targeted interventions. Addressing these issues through education, mental health support, and regulated internet use could mitigate the negative impacts on adolescents’ well-being.

**Disclosure of Interest:**

None Declared

